# Mitotic Arrest-Deficient 2 Like 2 (MAD2L2) Interacts with *Escherichia coli* Effector Protein EspF

**DOI:** 10.3390/life11090971

**Published:** 2021-09-15

**Authors:** Amin Tahoun, Hanem El-Sharkawy, Samar M. Moustafa, Lina Jamil M. Abdel-Hafez, Ashraf Albrakati, Manfred Koegl, Juergen Haas, Arvind Mahajan, David L. Gally, Ehab Kotb Elmahallawy

**Affiliations:** 1 Division of Immunity and Infection, The Roslin Institute and R(D)SVS, The University of Edinburgh, Easter Bush, Midlothian EH25 9RG, UK; a.kumarmahajan@elanco.com (A.M.); dgally@ed.ac.uk (D.L.G.); 2 Department of Animal Medicine, Faculty of Veterinary Medicine, Kafrelsheikh University, Kafrelsheikh 33511, Egypt; 3 Department of Poultry and Rabbit Diseases, Faculty of Veterinary Medicine, Kafrelsheikh University, Kafrelsheikh 33511, Egypt; hanem_amin@yahoo.com; 4 Department of Zoonoses, Faculty of Veterinary Medicine, Benha University, Benha 13511, Egypt; samar.mustafa@fvtm.bu.edu.eg; 5 Department of Microbiology and Immunology, Faculty of Pharmacy, October 6 University, October 6 City 12566, Giza, Egypt; Lina.jamil@ymail.com; 6 Department of Human Anatomy, College of Medicine, Taif University, P.O. Box 11099, Taif 21944, Saudi Arabia; a.albrakati@tu.edu.sa; 7 Preclinical Target Development and Genomics and Proteomics Core Facilities, German Cancer Research Center, 69120 Heidelberg, Germany; m.koegl@dkfz-heidelberg.de; 8 Division of Pathway Medicine and Centre for Infectious Diseases, University of Edinburgh, Edinburgh EH16 4SB, UK; juergen.haas@ed.ac.uk; 9 Department of Zoonoses, Faculty of Veterinary Medicine, Sohag University, Sohag 82524, Egypt

**Keywords:** MAD2L2, interaction, effector proteins, EspF, *Escherichia coli*, pathogenesis

## Abstract

*Enteropathogenic* (EPEC) and *Enterohemorrhagic* (EHEC) *Escherichia coli* are considered emerging zoonotic pathogens of worldwide distribution. The pathogenicity of the bacteria is conferred by multiple virulence determinants, including the locus of enterocyte effacement (LEE) pathogenicity island, which encodes a type III secretion system (T3SS) and effector proteins, including the multifunctional secreted effector protein (EspF). EspF sequences differ between EPEC and EHEC serotypes in terms of the number and residues of SH3-binding polyproline-rich repeats and N-terminal localization sequence. The aim of this study was to discover additional cellular interactions of EspF that may play important roles in *E. coli* colonization using the Yeast two-hybrid screening system (Y2H). Y2H screening identified the anaphase-promoting complex inhibitor Mitotic Arrest-Deficient 2 Like 2 (MAD2L2) as a host protein that interacts with EspF. Using LUMIER assays, MAD2L2 was shown to interact with EspF variants from EHEC O157:H7 and O26:H11 as well as EPEC O127:H6. MAD2L2 is targeted by the non-homologous *Shigella* effector protein invasion plasmid antigen B (IpaB) to halt the cell cycle and limit epithelial cell turnover. Therefore, we postulate that interactions between EspF and MAD2L2 serve a similar function in promoting EPEC and EHEC colonization, since cellular turnover is a key method for bacteria removal from the epithelium. Future work should investigate the biological importance of this interaction that could promote the colonization of EPEC and EHEC *E. coli* in the host.

## 1. Introduction

*Enteropathogenic* and *Enterohemorrhagic Escherichia coli* (EPEC and EHEC, respectively) constitute a significant major risk to human health worldwide [1]. In accordance with their public concern, EPEC are a subgroup of medically important diarrhoeagenic *E. coli* that can cause severe infantile diarrheal diseases in the developing world, arising mainly from human-to-human infection [2,3]. Furthermore, EPEC cause a serious intestinal disease in many animal species, including cattle [4,5,6]. On the other hand, EHEC are a class of Shiga-toxigenic *E. coli* (STEC) that are considered as a cause of potentially fatal foodborne infections, arising from animal-to-human infection, where cattle are considered the main reservoir host [7,8,9]. These emerging zoonotic pathogens are primarily found in industrialized countries and cause sporadic outbreaks of severe disease in humans, including hemorrhagic colitis (HC) and hemolytic uremic syndrome (HUS) [10]. Among others, *E. coli* O157:H7 is the predominant EHEC serotype associated with human disease in industrialized countries and recently is considered a leading cause of HUS [5,11,12,13]. Both EPEC and EHEC are defined by their capacity to adhere to host gastrointestinal cells and inject effector proteins, including their own anchoring and signaling receptor, via a type III secretion system (T3SS) that is encoded by the chromosomal locus of enterocyte effacement (LEE), a pathogenicity island that encodes many virulence factors important for EPEC and EHEC pathogenicity [14,15]. EspF, a LEE-encoded effector protein, translocates into host cells in a T3SS-dependent manner. Several previous reports have demonstrated that EspF disrupts tight junctions, which is followed by a series of events including increasing mitochondrial membrane permeabilization and cell death, inhibition of bacterial uptake by macrophages, and restriction of translocation across antigen-sampling M cells in the gut [16,17,18]. Furthermore, EspF is involved in the disruption of tight junctions and increases monolayer permeability through the redistribution of the intermediate filament protein CK18, rearranging the actin cytoskeleton, and interacting with Arp2/3, ZO-1, ZO-2 and occludins [16,19,20,21]. Importantly, several previous reports have shown that EspF sequences differ among EPEC and EHEC strains. Furthermore, the EspF sequence of the EHEC O157 variant has a significant impact on transepithelial electrical resistance (TER) [20]. In addition, EspF, in combination with other effectors, plays a major role in colonization of *E. coli* through the inhibition of the water transporter SGLT-1 [22]. EspF_EPECO127_ targeted the mitochondria, with the N-terminal region of EspF functioning as an import signal that accelerates targeting through the mitochondrial membrane protein Tom20 [23,24]. Furthermore, EspF_EPECO127_ induces mitochondrial membrane permeabilization that is associated with increased release of cytochrome C from mitochondria into the cytoplasm and subsequent caspase-9 and caspase-3 cleavage, which, in turn, targets the mitochondrial apoptosis pathway and cell death [16,19,24]. EspF interacts with Abcf2 and inhibits its anti-apoptotic effect, promoting cell apoptosis [19]. A previous study reported that EspF, in the later stage of infection, targets the nucleolus and leads to the loss of nucleolin from the nucleolus into the cytoplasm and a reduction in the level of the ribosomal protein RPL9 [25]. Moreover, EspF_EPECO127_ inhibited the phosphatidyl inositol-3 (PI3) kinase-dependent pathway of bacterial uptake, which represents an important role in *E. coli* pathogenesis [26]. A recent study demonstrated that EHEC O157:H7 EspF interacts with Annexin A6 (Anxa6), which is involved in many biological processes including cell proliferation, survival, differentiation, and inflammation, which may therefore play an important role in enhanced O157 persistence [27]. EspF has been shown to function as hijack host endosomes at bacterial adherence sites. This is considered as an additional adaptation to facilitate endocytosis and recycling of plasma membrane proteins at these sites early upon infection. This results in uptake of iron by bacteria that is considered a critical element for bacterial growth and virulence [28]. EspF was shown to be essential for infection in vivo using the mouse pathogen Citrobacter rodentium (encoding 31 effectors). Sequential gene deletions showed that EspF and Map effectors are essential for infection by maintaining pathogenicity and inducing IL22 secretion and protective immunity to prevent secondary infection [29]. EspF may mediate DNA damage by regulating the subcellular localization and phosphorylation of SMC1 [30]. MAD2L2 is necessary for cellular mitosis, cell cycle checkpoint, DNA damage response (DDR) and most commonly associated with normal physiological processes [31,32,33,34,35,36]. The process by DDR components results in a unique strategy to control cancers [33,34,35,36]. EspF is a multifunctional protein that interacts with several host cell signaling proteins. For example, EspF_EPECO127_ can bind to Sorting Nexin 9 (SNX9) and neuronal Wiskott–Aldrich syndrome protein (N-WASP) via its SH3 amino terminal region that plays a critical role in endocytosis and phagocytosis [37]. This binding between EspF and N-WASP induces actin polymerization and maturation of the actin-rich pedestals, promoting the colonization of pathogenic *E. coli* [21,38,39].

SNX9 is one of these recognized host proteins that, along with N-WASP, plays a vital role in endocytosis and phagocytosis. In previous work, we used LUMIER binding assays and demonstrated differences in the interactions of EspF variants with SNX9 and N-WASP [18]. The results of the LUMIER assays showed that both the EPEC O127 and EHEC O26 EspF variants had a greater capacity to interact with SNX9 and N-WASP than the EspF O157 variant. The result may explain the lower ability of the EspF O157 variant to prevent phagocytosis and inhibit *E. coli* translocation via a human-derived in vitro M-cell co-culture system in comparison to EspFO127 and EspFO26. However, it is clear that EspF has been shown to interact with a myriad of host proteins, and there are potentially other host proteins involved that have not been identified to date. The work presented here aimed to identify additional host proteins that interact with the effector protein EspF from EHEC and EPEC. This information would deepen our understanding of the mechanisms involved in the different responses to *E. coli* types in host cells that might trigger therapeutic targets to intervene and limit *E. coli* pathogenicity.

## 2. Materials and Methods

### 2.1. Gateway Cloning for Screening EspF Protein–Protein Interactions

This step aimed to simplify construction of vectors for Y2H analysis of EspF interactions with eukaryotic proteins. *espF* alleles were amplified using proofreading, using an expand long-template PCR system (Roche Diagnostics), using a set of primers, which are shown in Table 1, then cloned into the Gateway™ vector pDONR-207 (Table 2) using BP Clonase (Invitrogen, California, USA) to form entry clones. Clones were checked for the presence of the correct insert by DNA plasmid extraction and restriction digests using *Ban*II. Once in the Gateway™ system, clones were transferred to the destination vectors including pTREX-DEST30 (protein A fusions) and p*Renilla* (*luciferase fusions*).

The recombination reactions (BP reactions) were performed as described elsewhere with slight modification [18]. Reactions were incubated overnight at room temperature. One microliter of each reaction was transformed into DH5α competent cells, which were allowed to recover in SOC medium for 1–2 h at 37 °C, then plated on LB plates containing 15 μg/mL gentamycin and incubated overnight at 37 °C. The obtained clones were then checked for the expected insert by plasmid extraction and digestion using *Ban*II.

#### Gateway Cloning of Different espF Alleles into the pDONR 207 Vector

**Figure 1:**
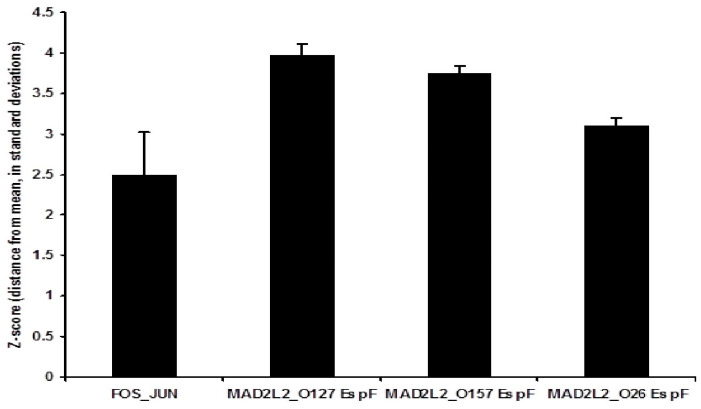
Comparative analysis of binding between EspF variants and human MAD2L2. In the LUMIER binding assay, the *espF* alleles were expressed with a protein A tag and then immobilized on immunoglobulin beads, while MAD2L2 was expressed with Renilla and incubated with the immobilized EspF variants (EspFO157, EspFO26, and EspFO127). Inverse combinations were also tested. Luminescence signals were then measured and z-scores calculated by subtracting the population mean from the individual raw score and then dividing the difference by the population standard deviation, FOS Jun was used as a control positive. MAD2L2 was confirmed to interact with all EspF variants (EspFO157, EspFO26, and EspFO127). The EspF O26 variant demonstrated the lowest interaction score of the three EspF binding to MAD2L2.

*espF* alleles were amplified using primers 1 and 2 for EHEC O26:H11 *espF* and primers 3 and 4 for EPEC and EHEC O157:H7 *espF* (Table 1). The PCR products were purified and then cloned into a donor vector according to the manufacturer’s instructions to form entry clones. Cloning into the pDONR 207 vector was initially confirmed by restriction digestion with *Ban*II (Figure 1). MAD2L2 and Nuclear Factor I C (NFIC) were initially cloned into the pDONR 223 vector and confirmed by digesting the vector with *Xba*I and *Nhe*I enzymes and sequencing.

### 2.2. Creation of Expression Clone via LR Reactions

Expression clones can be created by excision, integration, and recombination of entry clone containing the PCR products by taking advantage of recombination sites using the LR clonase complex. Destination vectors contain the *ccdB* gene between the left and right recombination sites. The LR reaction was carried out using the destination vector (300 μg/µL), entry clone (150 ng/µL), dH_2_O and LR clonase enzyme mix (Int, IHF, and Xis). Reactions were incubated for 3–4 h at room temperature, and 2 μL of each reaction product were transformed into DH5α chemical competent cells, which were allowed to recover on SOC medium for 1–2 h at 37 °C and then plated on LB plates containing the appropriate antibiotic and incubated overnight at 37 °C. The resulting clones were then checked for the expected insert by plasmid extraction and restriction digests (*Eco*RI and *Bam*HI for Bait and eGFP and *Xba*I and *Hind*III for His and Gst fusion construct plasmids).

#### 2.2.1. Sub-Cloning of espF from Entry Clones into Multiple Destination Vectors

*espF* expression clone was easily transferred into an attP pDONR vector (via the BP reaction) to generate an entry clone and then into different destination vectors, all without the need for restriction enzymes and ligase.

#### 2.2.2. Plasmids for Y2H and Colonization

*espF* entry clones were sub-cloned into the Y2H bait vector pGBKT7-DEST and the eukaryotic entry clone library sub-cloned into the prey vector pGADT7-DEST. Both vectors are destination vectors, with pGADT7-DEST encoding ampicillin resistance and pGBKT7-DEST encoding kanamycin resistance. The prey and bait clones were confirmed by restriction digests with *EcoR*I and *BamH*I (Figure S1).

#### 2.2.3. Plasmids for LUMIER Pull-Down Assays

A subset of EspF variants and the eukaryotic interaction partner MAD2L2 were sub-cloned from pDONR207 or pDONR223 into the LUMIER vectors pcDNA-Renilla and pT-REx-A to validate interactions (Figures S1 and S2, respectively). Both vectors are expression vectors that also confer ampicillin resistance. The Renilla-tagged clone sequences were confirmed by digesting with *Xho*I and *Xba*I, and the protein A-tagged clones were confirmed by restriction digests using *Xho*I and *Nhe*I (Figures S1 and S2).

#### 2.2.4. Plasmids for Transient Eukaryotic Expression and Validation of Y2H-Positive Interaction

**Figure 2:**
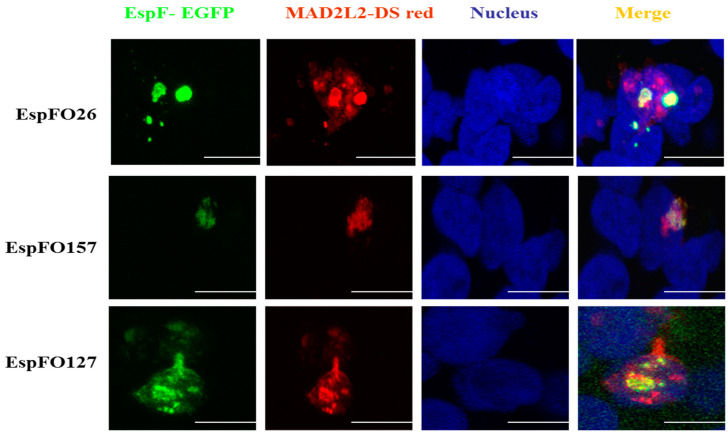
Confocal microscopy to validate the interaction of EspF and MAD2L2. Caco-2 cells were transfected with the expression plasmid peGFP-DEST (Promega)-expressing EspF tagged with GFP (green) and pDSRED2-DEST (Promega)-expressing DSred-tagged MAD2L2 (red), the co-localizations indicated by yellow color. After 48 h, cells were fixed and permeabilized with 2% (*w*/*v*) formalin/0.25% (*v*/*v*) Triton X-100. The cells were then incubated with DAPI to stain the nucleus (blue). Slides were mounted in fluorescence mounting medium (DAKO) and images acquired using a Leica TCS NT confocal system with a 63× objective. Scale bar 10 μm.

In order to examine the possible co-localization of EspF with eukaryotic protein MAD2L2 in vitro, *espF* entry clones were sub-cloned into peGFP-DEST vectors containing N-terminal GFP tags and kanamycin-resistant cassettes. Expression clones were confirmed by restriction digests using *EcoR*I and *BamH*I. MAD2L2 and entry clones were sub-cloned into pDSRED2-DEST containing an N-terminal PD tag and kanamycin-resistant cassettes and confirmed by restriction digests with *Xho*I and *Nhe*I (Figure 2).

### 2.3. Y2H System for Detecting Protein–Protein Interactions 

The Y2H system was first described by Fields and Song in 1989 [40], and is currently used to detect protein–protein interactions in eukaryotic cells. Y2H assays involve a transcription factor consisting of a DNA-binding domain (DNA-BD) that binds to a promoter and an activation domain that recruits the transcription complex. These domains are not functional on their own; however, when they are brought together, the activity of the transcription factor could be restored [40].

### 2.4. Direct Mating for Y2H Screens

Yeast bait and prey clones were streaked out from glycerol stocks on selective agar plates and incubated for 1–3 days at 30 °C. Haploid yeast cells were inoculated in 5 mL liquid medium for pre-cultures and then 5 mL of freshly grown yeast inoculated in 25 mL of singly selective liquid medium, either SD-Trp for bait or SD-Leu for prey. Cultures were incubated overnight at 30 °C and 220 rpm in an appropriate vessel that prevented cells from growing at high densities. Next, 100 µL of mating media (SD-Leu-Trp with 5% YPDA) were added to the required number of wells of a U-bottom microtiter plate, with a U-bottom plate used to ensure close proximity of yeast cells. Bait and prey cultures were resuspended in mating media to an OD_600_ of 1.0–1.2 and 25 μL of resuspended culture and transferred to each appropriate well of the mating plate. The plate was then centrifuged at 2000 rpm for 30 s at room temperature to bring cells in close proximity to each other at the bottom of each well to facilitate mating. The plate(s) were incubated at 30 °C overnight. The wells of a flat-bottom microtiter plate were filled with 150 μL of fresh double-selective media (SD-Leu-Trp containing penicillin/streptomycin) for diploid selection. Yeast pellets in the mating plate were resuspended and 10 μL transferred from each culture to the diploid selection plate and incubated at 30 °C without shaking for 2 days. Diploid cells were then checked visually for growth until saturation or near saturation were reached. Triple-knockout SD media (with no Trp, Leu, or His) containing the fluorescent detection compound 4-MuX and the bait auto-activation inhibitor 3-AT was then prepared. Duplicate assay plates were prepared by adding 150 µL of detection media (detection plate) or SD-LW (control diploid selection plate) to a sufficient number of wells of flat-bottom microtiter plates. Diploid cultures were suspended in the diploid selection plate and 10 μL of each culture transferred to the detection and control plate, covered with gas-permeable lids, and placed in plastic bags, then incubated at 30 °C for 3–7 days, with no shaking. Cells were suspended in the control plate and absorbance measured at OD_600_ relative to media-only negative controls to confirm yeast cell growth and mating success. The fluorescence readout was measured using a fluorescence reader, with excitation at 365 nm and emission at 448 nm.

### 2.5. LUMIER Assays

For the LUMIER assays, proteins were either fused with *Staphylococcus aureus* protein A or *Renilla reniformis* luciferase was fused at their amino termini before being transiently expressed in Caco-2 cells. Each expression construct (20 ng) was transfected into Caco-2 cells using 0.05 µL of lipofectamine 2000 (Invitrogen) in 96-well plates. After 40 h, the media were removed, and the cells were lysed on ice in 10 µL of ice-cold lysis buffer (20 mM Tris pH 7.5, 250 mM NaCl, 1% Triton X-100, 10 mM EDTA, 10 mM DTT, Protease Inhibitor Cocktail (Roche), Phosphatase Inhibitor Cocktail (Roche), and 25 units/µL Benzonase (Novagen)). Sheep-anti-rabbit IgG-coated magnetic beads were then added (Invitrogen, Dynabeads M280, 2 mg/mL final concentration) and the mixture was incubated on ice for 15 min. Next, 100 μL of washing buffer (PBS, 1 mM DTT) were added to each well and 10% of the diluted lysate removed to determine the luciferase activity present in each sample before washing. The rest of the sample was washed 6 times in washing buffer, and luciferase activity was measured in both lysate-washed beads. Negative controls consisted of wells transfected with plasmids expressing a luciferase fusion protein and protein A dimers were included. Negative controls were wells transfected with the plasmid expressing the luciferase fusion protein and a vector expressing a dimer of protein A. Normalised interaction signals were z-transformed by subtracting the mean and dividing by the standard deviation. The mean and standard deviation were calculated from large datasets of protein pairs which were expected to interact, i.e., from negative reference sets.

### 2.6. Transfection of Adherent Caco-2 Cells Using Effectene

The enhancer was used first to condense DNA; then, the Effectene reagent (Qiagen, UK) was used to coat the condensed DNA molecules with cationic lipids. Cells were plated in wells of 48-well plates at a density of 5 × 10^4^ cells/well in 400 µL DMEM with 10% (*v*/*v*) fetal calf serum (FCS, Sigma, Dorset, United Kingdom), 1 U of penicillin (Invitrogen, Paisley, United Kingdom), 1 µg/ml of streptomycin (Invitrogen, Paisley, United Kingdom) and 2 mM of L-glutamine (Invitrogen, Paisley, United Kingdom) 48 h before transfection in order to reach optimal transfection conditions of 40–70% confluence (approximately 2 × 10^5^ cells/well). Total DNA (150 ng) was then diluted in EC buffer to a final volume of 50 and 1.2 µL of enhancer, after which the solution was mixed by vortexing and incubated for 5 min at room temperature to allow for formation of condensed DNA. Next, 4 µL of Effectene transfection reagent were added to the aforementioned solution and incubated for 10 min at room temperature for DNA transfection complex formation. Cell media were then removed from each plate, cells washed with PBS, and 200 µL of fresh DMEM containing serum and antibiotics added to each well. After lipid–DNA complex formation, 200 µL of fresh DMEM containing serum and antibiotics were then added to the DNA mixture, which was then placed onto adherent cells drop by drop and swirled to ensure an even distribution. Protein expression levels were determined 48–72 h post-transfection. The transfection efficiency was determined after 24 h using eGFP expression as a positive readout.

### 2.7. Confocal Microscopy

Caco-2 cells grown on chamber glass slides were transfected with N-terminally GFP-tagged EspF expressed from pAT11-13 and DSred-tagged MAD2L2 expressed from pAT20 (Table 2). The cells were either fixed in 2% (*v*/*v*) Formalin or fixed/permeabilized in 2% (*v*/*v*) Formalin-0.2% (*v*/*v*) Triton X-100 for 20 min at room temperature. Cells were then treated for 20 min at room temperature with 1/5000 DAPI (Molecular Probes), washed twice with PBS and mounted in fluorescent mounting medium (DAKO). The confocal data were acquired using a Zeiss Plan Apochromat 1.4 NA with a 63× oil immersion lens and a multi-track (sequential scan) experimental setup on a Zeiss LSM510 with images taken at a pixel image size of 1024 × 1024. Image data were acquired at Nyquist sampling rates and deconvolved using Huygens software (Scientific Volume Imaging, Laapersveld 63, 1213 VB Hilversum, The Netherlands). The resulting three-dimensional models were analyzed, and orthogonal views were created using NIH ImageJ software, with final figures assembled using Adobe Photoshop. The colocalization of the percentage of MAD2L2 and EspF variants was performed using ImageJ software.

## 3. Results

### 3.1. MAD2L2 Is a Novel EspF Interacting Partner

In previous work, we have confirmed that EspF interacts with SNX9 and N-WASP, confirming its critical role during infection [18]. Furthermore, we have shown differences between EPEC and EHEC resistance to phagocytosis that was attributed to the interaction of their EspF variants with SNX9 and N-WASP using LUMIER binding assays [18]. In order to identify more cellular ligands that might interact with this type 3 effector protein, EspF variants were screened against a human cDNA library using the Y2H system [41]. The mating between the bait strain, containing EspF variants, and the prey strain, containing cDNA for a human protein library, suggested a new protein hit for EspF, with an interaction between EspF from EHEC O26:H11 and the host MAD2L2 protein. In stark contrast, no interaction was detected between EspF and EPEC O127:H6.

### 3.2. Binding Activity of the Three EspF Variants to MAD2L2

In order to verify the results of the Y2H screen, and to investigate the differential quantitative levels of the binding activity of the three EspF variants to human MAD2L2 protein, LUMIER binding assays were carried out. MAD2L2 and EspF variants (EspFO157, EspFO26, and EspFO127) were cloned into plasmids that expressed either protein A-tagged proteins or Renilla luciferase fusion proteins and vice versa. These plasmids were then transiently expressed in Caco-2 cells. Negative controls consisted of wells transfected with plasmids expressing a luciferase fusion protein and protein A dimers were also used [42]. Cell lysates containing both sets of tagged proteins were generated and then the protein A-tagged complexes were removed using antibody-coated beads. The total fluorescence and captured fluorescence were measured and z-scores for each interaction calculated by comparison with a large bank of negative non-interacting controls. Interestingly, we found that all EspF variants (EspFO157, EspFO26, and EspFO127) interacted with MAD2L2 and these quantitative results demonstrated that EspFO26 had the lowest binding affinity to MAD2L2 of the three investigated variants, but this difference was not statistically significant (Figure 1). The interaction between EspF variants with MAD2L2 suggests that all three bacterial EspF have the ability to bind and activate MAD2L2.

### 3.3. Demonstration of the Intracellular Interaction of EspF and MAD2L2

**Figure 3:**
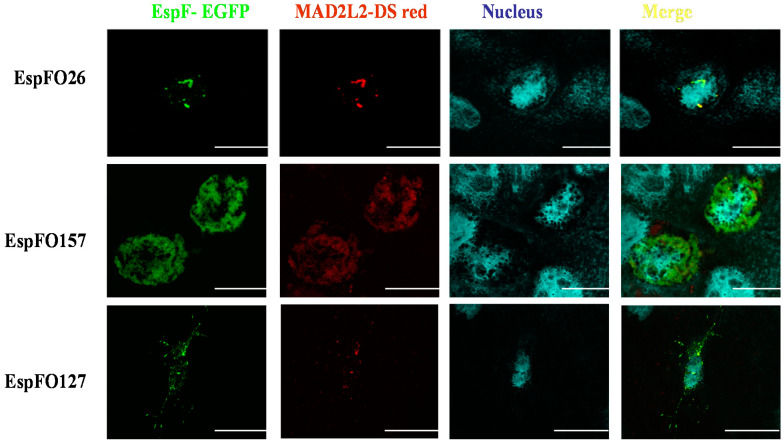
Fluorescence microscopy images showing EspF (green), MAD2L2 (red), and their co-localization in the nucleus (yellow). A sub-confluent monolayer of Caco-2 epithelial cells was transfected with a Red Fluorescent Protein (DSred) labelled MAD2L2 (red) and GFP labelled EspF for 72 h. To examine the binding, their co-localization, formation of physical binding (yellow) were examined by immunofluorescence. The presence of vacuolations in the nuclei indicates the apoptosis caused by EspF. Images acquired using a Leica TCS NT confocal system with a 63× objective. Scale bar 10 μm.

Caco-2 epithelial cells were double-transfected with N-terminally GFP-tagged EspF expressed from pAT11-13 and DSred-tagged MAD2L2 expressed from pAT20 to confirm that different EspF variants are able to bind to MAD2L2 and examine the relative intracellular distribution of these protein complexes. Confocal microscopy examination of the transfected cells revealed that, at 48 h, all the generated EspF protein, irrespective of EspF variant, co-localized with MAD2L2 in the cell nuclei, which did not yet show any sign of cell apoptosis (Figure 2). Extending the incubation time to 72 h before fixation still found a distinct co-localization of EspF variants with MAD2L2 inside the nucleus of each transfected cell. However, cellular vacuolation was observed, suggesting that apoptotic cell death had been induced (Figure 3). We determined the percentage of co-localization by dividing the area of co-localization by the total fluorescence of the whole cell. We also found that the percentage of co-localization of the EspF variants, EspFO157, EspFO26, and EspFO127, with MAD2L2 was 21.76% ± 2.2, 19.85% ± 0.65, and 28.87% ± 2.73, respectively, and there was no significant difference between each other using ANOVA. These results correlate with the quantitative LUMIER binding studies (Figure1) and further confirm the interaction between EspF variants and MAD2L2 protein inside the nuclei. In addition, the present results suggest a mechanism behind how EspF increases the ability of different pathogenic *E. coli* to reinforce their colonization inside epithelial cells through inactivation of MAD2L2 protein, which, in turn, leads to activation of the anaphase-promoting complex APC/cyclosome, thus inhibiting epithelial cell turnover and reducing *E. coli* removal from the site of infection.

## 4. Discussion

Elucidation of the molecular mechanisms behind the pathogenesis of EHEC and EPEC is important since they are a major cause of human diseases [43,44]. Diarrhoea in infants and young children is mainly caused by EPEC [45], while EHEC infection leads to mortality worldwide through acute renal failure and hemolytic uremic syndrome [43]. It is noteworthy to state that both EPEC and EHEC persist in human or animal hosts through attachment to epithelial cells that line the gastrointestinal tract [4]. These pathogenic bacteria use Type III secreted effector proteins to manipulate the actin cytoskeleton and innate responses of host cells to enable intimate bacterial attachment and persistence [46]. T3SS-induced translocation of the effector protein EspF results in tight junction disruption, water channel dysregulation and inhibition of bacterial uptake into M cells [17,39]. The EspF effector protein has been demonstrated to localize in both the host cell mitochondria and nuclei [16,18,25] and is known to interact with SNX9, N-WASP, and ABCF2 [19,38]. This current study presents interesting data concerning the molecular mechanism by which pathogenic *E. coli* may promote their colonization of intestinal epithelial cells, supporting the role of EspF interacting with the cell cycle protein MAD2L2. To elucidate this protein–protein interaction, we implemented a Y2H screening system, which is considered an excellent evolving system for high-throughput screening of protein–protein interactions [41]. In previous work, we established that there are differences between the ability of EPEC and EHEC strains to resist phagocytosis by macrophages and transcytosis by M-like cells, which we attributed to variation in the interaction of their different EspF proteins with SNX9 and N-WASP using LUMIER binding assays [18].

In the present study, the Y2H system was used to screen EspF bait clones against a human cDNA library prey, which resulted in a successful mating between bait strain containing EspF and the prey strain containing cDNA library protein, identified as MAD2L2, and the reconstitution of transcription factor activity that further indicated a protein–protein interaction. This bait-prey interaction-induced transcription factor activity led to the expression of the easily detectable reporter gene producing the characteristic fluorescence. Previously, the molecular mechanisms by which *E. coli* T3SS-effector protein EspF induced apoptosis in the intestinal epithelial barrier of the host were shown to be indistinct [47]. The results of this current study have begun to answer this important question. Using LUMIER assays, we confirmed the ability of EspF variants from EHEC O157:H7 and O26:H11, as well as EPEC O127:H6, to interact with the anaphase-promoting complex (APC) inhibitor; MAD2L2. Taken together, these studies suggest a novel mechanism by which EspF can slow epithelial cell turnover and confer an advantage for prolonged bacterial colonization [48]. It is noteworthy to state that confocal microscopy has been used to confirm co-localization of EspF and MAD2L2 inside the nuclei of epithelial cells. The binding of EspF variants with MAD2L2 after 48 h inside the nuclei of Caco-2 cells is in agreement with a similar recent finding in HEK293 cells [49], again without inducing cell apoptosis (Figure 2). However, by 72 h, cells co-expressing EspF and MAD2L2 showed the characteristics of apoptosis (Figure 3), suggesting that this protein–protein interaction is inhibiting the cell cycle [50]. This novel identification of MAD2L2 as a target of the EHEC and EPEC effector protein EspF is in line with a previous report that MAD2L2 is targeted by the non-homologous *Shigella* effector protein IpaB to limit epithelial cell turnover [48]. This interaction of EspF with the cell cycle key player MAD2L2 would be expected to greatly affect the cell cycle and confer an advantage for bacterial survival.

The MAD2-related protein MAD2L2 is an inhibitor of the APC Cdh1 [51,52,53]. Activation of the APC during interphase (G1/S) leads to cell cycle arrest at G2/M. The interaction between Cdh1 and MAD2L2 has been shown to result in the inhibition of Cdh1-APC complex activity; thus, MAD2L2 may play an important role as a mitotic spindle assembly checkpoint protein [53], preventing the onset of anaphase until all chromosomes are properly aligned at the metaphase plate. MAD2L2 also plays a pivotal role in translational DNA synthesis in the S phase [54] and, similar to MAD2L2, participates in the spindle assembly checkpoint [55]. MAD2L2 has been shown to bind to the TCF4-catenin forming interaction complex that inhibits differentiation to Epithelial mesenchymal transition (EMT) and enhances proliferation of epithelium cells [56,57]. *Shigella* IpaB was shown to sequester MAD2L2 away from the APC-Cdh1 complex, leading to unregulated APC activity during interphase and subsequent cell cycle arrest [48]. Cell division plays an essential role in innate immunity, as the rapid turnover of epithelial cells limits bacterial colonization. Therefore, slowing this turnover could increase the ability of bacteria to multiply and hence prolong bacterial colonization [48]. Clearly, the present results add new knowledge to our understanding of the molecular pathogenesis of EHEC and EPEC and their weapons to ensure efficient colonization inside the host epithelial cells. Based on this new detailed understanding, abolishing the EspF interaction with MAD2L2 could be a therapeutic target to limit bacterial colonization, pathogenicity and severity of the diseases caused by EHEC and EPEC.

## 5. Conclusions

The present study revealed the ability of EHEC and EPEC effector protein EspF to interact with and bind to MAD2L2. MAD2L2 normally inhibits the anaphase-promoting complex APC/cyclosome. The binding of EspF to MAD2L2 will free the APC, which stops the cell cycle progression and prevents epithelial cell turnover. As cellular turnover is a key method for bacteria removal from the epithelium, stopping MAD2L2 activity promotes bacterial colonization in the intestinal mucosa. Collectively, the present results added more depth to the understanding of EHEC and EPEC molecular pathogenesis and abolishing this reported interaction in future studies could be helpful in designing novel therapeutic targets for combating the diseases caused by EHEC and EPEC. Further future research is also warranted to address whether the translocated EspF into the cells following infection interacts with MAD2L2 and its co-localization, combined with exploring the possible involvement of this interaction on cell cycle and/or APC/C parameters.

## Figures and Tables

**Table 1:** List of primers used in this study.

Primer Name	Sequence	Primer Use
1. O26 *espF* gat fore 2. O26 *espF* gat rev	1. GGGG-ACA-AGT-TTG-TAC-AAA-AAA-GCA-GGC-T(CC-GCC) atgcttaatggaattagtcaagc 2. GGGG-AC-CAC-TTT-GTA-CAA-GAA-AGC-TGG ctacacaaaccgcatag	Gateway cloning of EHEC O26 *espF*
3. *espF*O157 and *espF*O127gat fore 4. *espF*O157 and *espF*O127 gat rev	3. GGGG-ACA-AGT-TTG-TAC-AAA-AAA-GCA-GGC-T(CC-GCC) atgcttaatggaattagtaacgc 4. GGGG-AC-CAC-TTT-GTA-CAA-GAA-AGC-TGG ctaccctttcttcgattgctcatag	Gateway cloning of *espF*O157 and *espF*O127

**Table 2:** List of plasmids used in this study.

Plasmids	Description	Source
pTREX-DEST30-prA	vector to generate amino terminus protein A fusions for LUMIER binding assays	laboratory stocks
pRenilla	Vector to generate amino terminus luciferase fusions for LUMIER binding assays	laboratory stocks
pAT6-7	*espF*O157, *espF*O26 and *espF*O127 cloned into pTREX-DEST30-prA, respectively	this study
pAT8-10	*espF*O157, *espF*O26 and *espF*O127 cloned into pRenilla, respectively	this study
pAT11-13	*espF*O157, *espF*O26 and *espF*O127 cloned into peGFP-DEST, respectively	this study
pHJ3	MAD2L2 in pDONR223	Gift from Prof. Juergen Haas, University of Edinburgh, UK
pAT18-20	pTREX-DEST30-MAD2L2, pRenilla-MAD2L2vectors expressing N-MAD2L2-protein A or -luciferase fusion proteins and MAD2L2 in DSred fusion plasmid pDSRED2-DEST	this study
pHJ4	NFICin pDONR2234	Gift from Prof. Juergen Haas, University of Edinburgh, UK
pAT21-23	pTREX-DEST30-NFIC, pRenilla-NFICvectors expressing NFIC-protein A or -luciferase fusion proteins and MAD2L2 in DSred fusion plasmid pDSRED2-DEST	this study

## Data Availability

The data that support the findings of this study are available on request from the corresponding author.

## Supplementary Materials

The following are available online at https://www.mdpi.com/article/10.3390/life11090971/s1, Figure S1: Gateway cloning of different *espF* alleles to study protein–protein interactions. Scanned image of gel red-stained 1% TAE agarose gel shows PCR amplification of the *espF* alleles from EPEC O127:H6 and the EHEC serotypes O157:H7 and O26:H11 (A). *Ban*II digests of different *espF* clones in the entry vector pDONR207 (B) and *EcoR*I and *BamH*I digests of different ORF clones in bait constructs (C). *EcoR*I and *BamH*I digests of different *espF* clones in the peGFP vector (C). *Xho*I and *Xba*I digests of different ORF clones in the pcDNARenilla vector (D). *Xho*I and *Nhe*I digests of different ORF clones in pTREX (D). Lane M contains the 1 kbp-plus DNA ladder (Invitrogen), and lanes 1–3 *espF* show results for *EPEC* O127:H6 and *EHEC* serotypes O157:H7 and O26:H11, respectively; Figure S2. Gateway cloning of Mad2L2 gene for protein–protein interaction studies. Scanned image of gel red-stained 1% TAE agarose gels showing results of XhoI and XbaI digests of mad2L2 clone in the entry vector pDONR223 (A), which were then cloned into two different destination vectors to create expression clones. EcoRI and BamHI digests of different ORF clones of bait (B) and prey (C) constructs. XhoI and XbaI digests of different ORF clones in the pcDNARenilla vector (D). XhoI and NheI digests of different ORF clones in pTREX (E). EcoRI and BamHI digests of different ORF clone into PDSred (F). Lane M contains the 1 kbp plus DNA ladder.
