# Supplementary material for: Mitotic Arrest-Deficient 2 Like 2 (MAD2L2) Interacts with Escherichia coli Effector Protein EspF
Source: Life (Basel). 2021 Sep 15;11(9):971. doi: 10.3390/life11090971 (PMC8469580; doi:10.3390/life11090971)
Supplement: Supplementary file 1 [file life-11-00971-s001.zip › life-1325852-supplementary.pdf]

life

Article

Mitotic Arrest-Deficient 2 Like 2 (MAD2L2) Interacts with *Escherichia coli* Effector Protein EspF

Amin Tahoun [1,2,*], Hanem El-Sharkawy [3], Samar M. Moustafa [4], Lina Jamil M. Abdel-Hafez [5], Ashraf Albrakati [6], Manfred Koegl [7], Juergen Haas [8], Arvind Mahajan [1], David L. Gally [1] and Ehab Kotb Elmahallawy [9,*]

[1] Division of Immunity and Infection, The Roslin Institute and R(D)SVS, The University of Edinburgh, Easter Bush, Midlothian EH25 9RG, UK; a.kumarmahajan@elanco.com (A.M.); dgally@ed.ac.uk (D.L.G.)
[2] Department of Animal Medicine, Faculty of Veterinary Medicine, Kafrelsheikh University, Kafrelsheikh 33511, Egypt
[3] Department of Poultry and Rabbit Diseases, Faculty of Veterinary Medicine, Kafrelsheikh University, Kafrelsheikh 33511, Egypt; hanem_amin@yahoo.com
[4] Department of Zoonoses, Faculty of Veterinary Medicine, Benha University, Benha 13511, Egypt; samar.mustafa@fvtm.bu.edu.eg
[5] Department of Microbiology and Immunology, Faculty of Pharmacy, October 6 University, October 6 City 12566, Giza, Egypt; Lina.jamil@ymail.com
[6] Department of Human Anatomy, College of Medicine, Taif University, P.O. Box 11099, Taif 21944, Saudi Arabia; a.albrakati@tu.edu.sa
[7] Preclinical Target Development and Genomics and Proteomics Core Facilities, German Cancer Research Center, 69120 Heidelberg, Germany; m.koegl@dkfz-heidelberg.de
[8] Division of Pathway Medicine and Centre for Infectious Diseases, University of Edinburgh, Edinburgh EH16 4SB, UK; juergen.haas@ed.ac.uk
[9] Department of Zoonoses, Faculty of Veterinary Medicine, Sohag University, Sohag 82524, Egypt
* Correspondence: amin12_veta@yahoo.com (A.T.); eehaa@unileon.es (E.K.E.)

Supporting Material

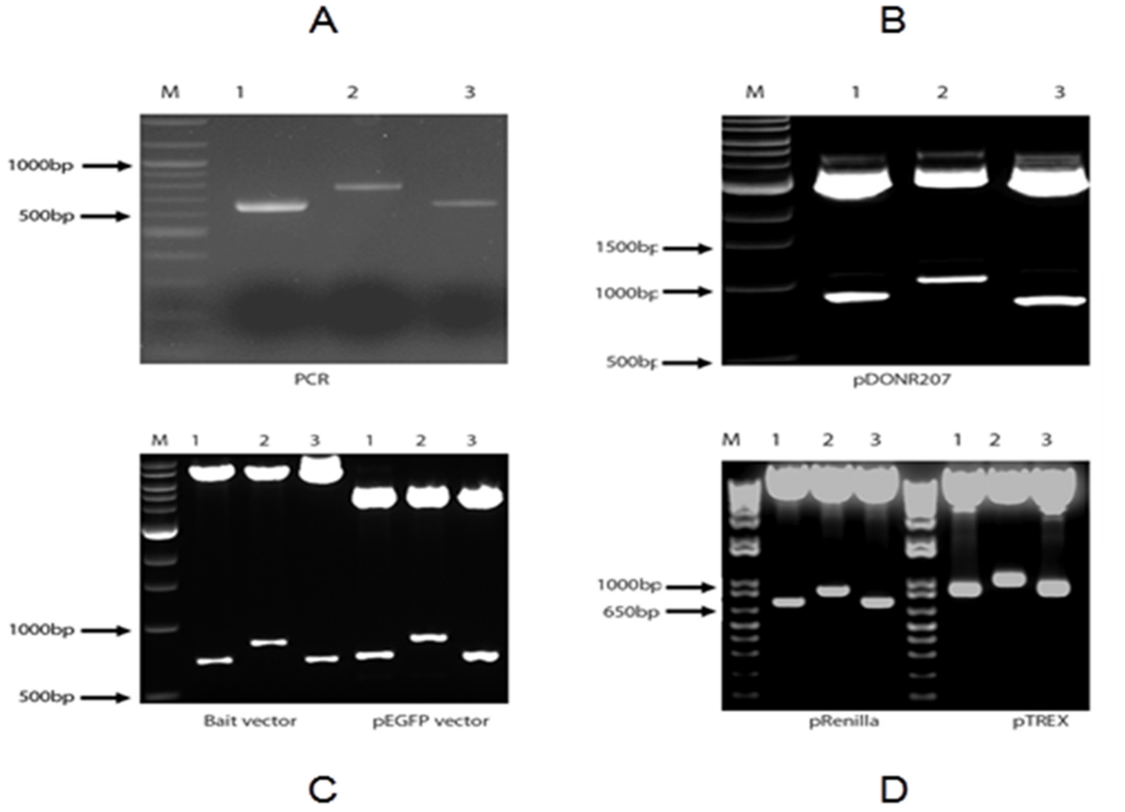

Figure S1. Gateway cloning of different *espF* alleles to study protein–protein interactions. Scanned image of gel red-stained 1% TAE agarose gel shows PCR amplification of the *espF* alleles from EPEC O127:H6 and the EHEC serotypes O157:H7 and O26:H11 (**A**). *Ban*II digests of different *espF* clones in the entry vector pDONR207 (**B**) and *EcoR*I and *BamH*I digests of different ORF clones in bait constructs (**C**). *EcoR*I and *BamH*I digests of different *espF* clones in the peGFP vector (**C**). *Xho*I and *Xba*I digests of different ORF clones in the pcDNARenilla vector (**D**). *Xho*I and *Nhe*I digests of different ORF clones in pTREX (**D**). Lane M contains the 1 kbp-plus DNA ladder (Invitrogen), and lanes 1–3 *espF* show results for *EPEC* O127:H6 and *EHEC* serotypes O157:H7 and O26:H11, respectively.

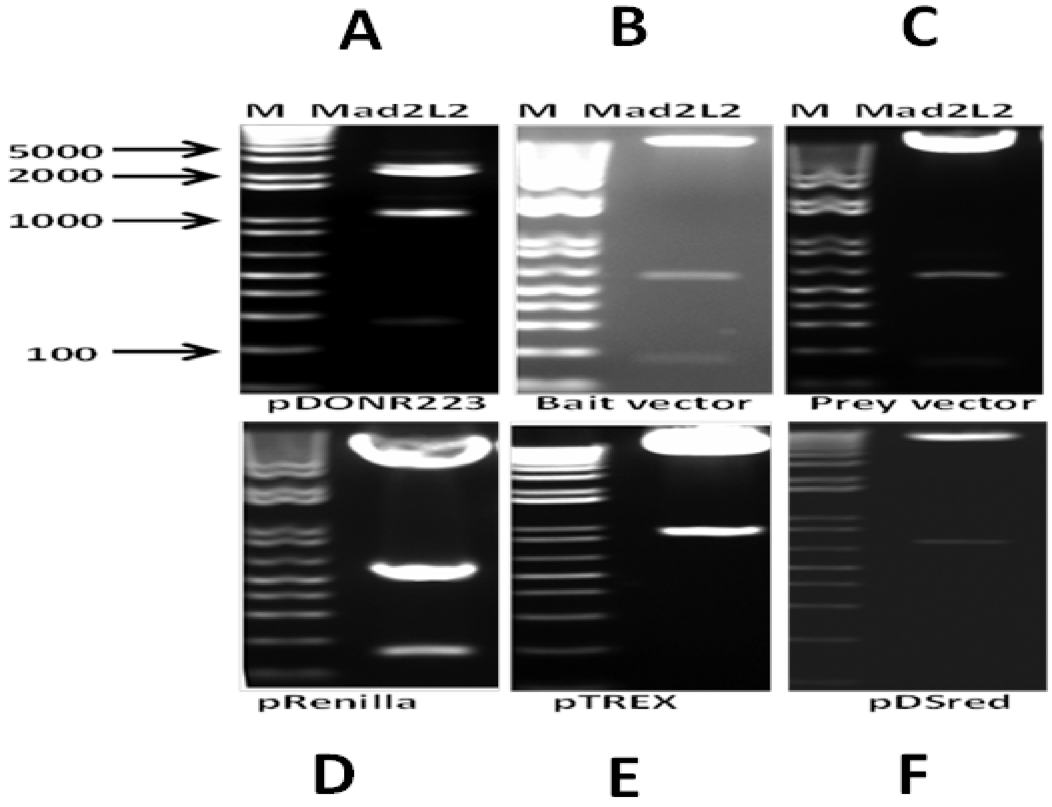

Figure S2. Gateway cloning of Mad2L2 gene for protein–protein interaction studies. Scanned image of gel red-stained 1% TAE agarose gels showing results of XhoI and XbaI digests of mad2L2 clone in the entry vector pDONR223 (**A**), which were then cloned into two different destination vectors to create expression clones. EcoRI and BamHI digests of different ORF clones of bait (**B**) and prey (**C**) constructs. XhoI and XbaI digests of different ORF clones in the pcDNARenilla vector (**D**). XhoI and NheI digests of different ORF clones in pTREX (**E**). EcoRI and BamHI digests of different ORF clone into PDSred (**F**). Lane M contains the 1 kbp plus DNA ladder.
